# Are sexual media exposure, parental restrictions on media use and co-viewing TV and DVDs with parents and friends associated with teenagers' early sexual behaviour?^☆^

**DOI:** 10.1016/j.adolescence.2013.08.019

**Published:** 2013-12

**Authors:** Alison Parkes, Daniel Wight, Kate Hunt, Marion Henderson, James Sargent

**Affiliations:** a Medical Research Council/Chief Scientist Office Social and Public Health Sciences Unit, 4 Lilybank Gardens, Glasgow G12 8RZ, United Kingdom; b Norris Cotton Cancer Center, Dartmouth Medical School, Lebanon, NH, USA

**Keywords:** Adolescent, Media, Sexual behaviour, Co-viewing

## Abstract

Sexual content in teenagers' media diets is known to predict early sexual behaviour. Research on sexual content has not allowed for the social context of media use, which may affect selection and processing of content. This study investigated whether sexual media content and/or contextual factors (co-viewing, parental media restrictions) were associated with early sexual behaviour using 2251 14–15 year-olds from Scotland, UK. A third (*n* = 733) reported sexual intercourse. In multivariable analysis the likelihood of intercourse was lower with parental restriction of sexual media and same-sex peer co-viewing; but higher with mixed-sex peer co-viewing. Parental co-viewing, other parental restrictions on media and sexual film content exposure were not associated with intercourse. Findings suggest the context of media use may influence early sexual behaviour. Specific parental restrictions on sexual media may offer more protection against early sex than other restrictions or parental co-viewing. Further research is required to establish causal mechanisms.

## Introduction

Early sexual initiation (before age 16) is likely to involve sexual risk-taking and expose young people to unwanted sex, sexually transmitted infections, and teenage pregnancy (Martinez, Copen, & Abma, 2011; Zimmer-Gembeck & Helfand, 2008). Teenagers in high-income countries are exposed to a highly sexualized media environment that may represent a primary source of sexual socialization (Ward, 2003; Wright, Malamuth, & Donnerstein, 2012). Sexual media content affects attitudes and beliefs conducive to early sexual initiation, including more permissive sexual norms, more positive expectations regarding sex and greater safe-sex self-efficacy (Bleakley, Hennessy, Fishbein, & Jordan, 2011; Braun-Courville & Rojas, 2009; Martino, Collins, Elliott, Kanouse, & Berry, 2009; Peter & Valkenburg, 2009). Several US longitudinal studies have found that exposure to sexual content in TV programmes, films, video games, magazines and music predicts earlier timing of sexual behaviour (Bleakley, Hennessy, Fishbein, & Jordan, 2008; Brown & L'Engle, 2009; Brown et al., 2006; Collins et al., 2004; Martino et al., 2006; O'Hara, Gibbons, Gerrard, Li, & Sargent, 2012). These studies allow for other well-established psychosocial influences on the timing of first sex, including family processes and friendships (Kotchick, Shaffer, Forehand, & Miller, 2001; Zimmer-Gembeck & Helfand, 2008). Yet capturing these important general influences may not adequately allow for the extent to which teenagers' experiences and interpretation of media are shaped by parents and friends. Media “use and gratification” theory stresses the importance of the social context for shaping audience orientation and involvement (Rubin, 1993). The extent of a teenager's exposure to sexual content could turn out to be less important for sexual behaviour than the social context of this exposure. Contextual factors, including parental mediation of media use and sharing media exposure with friends, may influence both the selection of sexual media content and how teenagers process and respond to this content.

Parental mediation of teenagers' media exposure involves any of three different behaviours that occur before, during and after media use and are distinct from other more general aspects of parent–child interactions, such as overall parental monitoring of the teenagers' leisure time (Nathanson, 2001a). Mediation behaviours may involve setting rules on how much, when and what media content teenagers can use (restrictive mediation), discussing media content with the teenager (active or instructive mediation), and the act of using media together, such as co-viewing TV (Valkenburg, Krcmar, Peeters, & Marseille, 1999).

Parental restrictions may lessen exposure to particular forms of media content. Restrictions may also reduce the extent to which children pay attention and attach importance to violent and sexual TV content (Nathanson, 1999) or reduce sensation-seeking (de Leeuw et al., 2011). However, one study of teenagers suggested parental restriction may also have unwanted consequences, encouraging less positive attitudes towards parents and more co-viewing with friends (Nathanson, 2002). A few studies of teenage television viewing have suggested parental restriction of TV is protective against early sex (Ashby, Arcari, & Edmonson, 2006; Bersamin et al., 2008; Fisher et al., 2009). Two of these studies (Bersamin et al., 2008; Fisher et al., 2009) measured parental restrictions in general terms, taking into account checks on what teenagers were watching, limits on duration of viewing and any prohibition of programmes. Another measured parental restrictions on content, but found a protective effect only for teenagers who also reported strong parental disapproval of sex (Ashby et al., 2006). It is not clear whether parental restrictions may have an independent protective effect regardless of a teenager's exposure to sexual content and other more general parenting processes already known to protect against early sex, such as supervision of free time (Buhi & Goodson, 2007; Zimmer-Gembeck & Helfand, 2008), since none of the studies of parental restriction took account of both these factors. It is also unknown whether particular aspects of parental restrictions, such as specific rules restricting sexual content, are more important than other restrictions on media use.

Despite the near universal prevalence of bedroom TV, internet access and music players for teenagers in many high-income countries (Bovill & Livingstone, 2001; Rideout, Foehr, & Roberts, 2010), most teenagers still prefer to watch TV in the living room where it provides opportunities for social interaction and discussion of programmes with parents (“active mediation”) (Adriaens, Van Damme, & Courtois, 2011; Nathanson, 1999). Without overt expression of parental disapproval, co-viewing may signal parental endorsement of programmes that parents and children watch together (Nathanson, 2001b, 2002). However, parental TV co-viewing and restrictions on media use both decline through the teenage years (de Leeuw et al., 2011; Sang, Schmitz, & Tasche, 1992). Lone viewing and peer co-viewing become more important, and the latter can strengthen peer relationships and group identity (Suess et al., 1998). Limited research on associations between parental co-viewing of TV and early sexual behaviour presents mixed findings, with one study reporting a protective effect (Bersamin et al., 2008) and another a null finding (Fisher et al., 2009). To date, however, there is very little research on the effects of co-viewing TV with friends on risk behaviours. One study found that peer co-viewing was associated with greater exposure to “antisocial” (sexual and violent) television content, greater peer discussion and approval of TV content and in turn predicted more aggressive behaviour on the part of the individual teenager (Nathanson, 2001c). In contrast, effects of parental mediation of TV were relatively weak. However, this study involved retrospective reporting by college students and did not allow for other more general family processes. To our knowledge, there has been no research investigating whether peer co-viewing is associated with early sexual behaviour.

The current study examines associations between different aspects of fifteen year olds' media environment and sexual behaviour in a large school-based sample, in order to investigate whether the amount and context of sexual media exposure may both influence the timing of first intercourse. Sexual media content is measured using exposure to sexual film content. Films are likely to form an important component of teenagers' media diet (Rideout et al., 2010; Wright et al., 2012) and their sexual content has already been linked to early sexual behaviour in a US longitudinal study (O'Hara et al., 2012). Other aspects of film content have also been associated with adolescent risk behaviours, for example depictions of smoking with teenage smoking (Dal Cin, Stoolmiller, & Sargent, 2012; Dalton et al., 2009; Sargent et al., 2007; Titus-Ernstoff, Dalton, Adachi-Mejia, Longacre, & Beach, 2008) and depictions of drinking with alcohol consumption (Hanewinkel & Sargent, 2009; Hanewinkel et al., 2012; Hanewinkel, Tanski, & Sargent, 2007; Sargent, Wills, Stoolmiller, Gibson, & Gibbons, 2006; Stoolmiller et al., 2012). Different types of parental restriction on media use are distinguished, to explore the question of whether a specific restriction on sexual content is more important than other types of restriction. We investigate whether there are protective effects of more frequent parental co-viewing television, both in absolute terms and relative to peer co-viewing. We also examine whether there are different effects of co-viewing with same-sex and with mixed-sex friends, with the hypothesis that mixed-sex viewing will be more strongly associated with sexual behaviour.

## Methods

### Data set

The study used a cross-sectional design. Data were collected over a two year period (2008–2009) as part of an evaluation of Healthy Respect Phase 2, a teenage sexual health programme (Elliott, Henderson, Nixon, & Wight, 2013). Pupils from seven secondary schools in the “intervention” area (East of Scotland) were given the SHARE sex education programme (Wight et al., 2002), and compared with pupils from seven schools in the West of Scotland (“comparison” area). Male pupils in the intervention area were more likely to use condoms than those in the comparison area, but otherwise the effects of the programme were largely confined to improved sexual health knowledge (Elliott et al., 2013). Anonymous confidential questionnaires were completed by 3981 pupils (88% of those eligible) aged 14–17 years under classroom examination conditions, supervised by researchers with no teachers present. Ethical permission was obtained from Napier University Ethics committee. Informed consent was sought from parents and pupils. 25 parents and 9 pupils refused participation.

### Main outcome measure

Sexual intercourse initiation was measured using the question: “Have you experienced the following with another person: penetrative sex?” where penetrative sex was defined as “both vaginal and anal sex and is intended to include both heterosexual sex and homosexual sex”.

### Main media exposure measures

Sexual film content in common with the only other study focusing exclusively on sexual film content (O'Hara et al., 2012), we used the Beach Method (Hunt, Henderson, Wight, & Sargent, 2011; Sargent, Worth, Beach, Gerrard, & Heatherton, 2008), although in our study this was modified to take account of repeat viewing of films. Films were selected from a sample of 463 US top box office hits, mainly from 2002 to 2008 (nine films made before 2002 were included in the list, based on responses to a question in an earlier survey about favourite films). Each pupil received a unique list of 50 films, using stratified random sampling with three strata defined by UK ratings as follows: U and PG (films deemed suitable for all children ‘Universal’ or for all with ‘Parental Guidance’); 12 and 12A (classified respectively as suitable only for those aged over 12 years, or under 12s only with an accompanying adult); 15- and 18-rated films (for those aged 15 and over, or 18 and over respectively). This allowed films to be sampled in proportion to the distribution of UK ratings in the box office sample (162 films were rated 15 and 18 with 30 of these being 18-rated, 159 were rated 12 and 12A and 142 rated PG and U). This meant that approximately one third of the 50 films each teenager was asked about were rated as 15 and 18, one third were rated 12 and 12A and a third rated PG and U.

Researchers examining associations between sexual film or TV content and teenagers' sexual behaviour have adopted various approaches towards content analysis, in terms of sampling strategy and coding of sexual behaviours and/or talk directed at sex (Bleakley et al., 2008; Brown et al., 2006; Collins et al., 2004; O'Hara et al., 2012). Again in common with another study focusing on sexual film content (O'Hara et al., 2012), the total sexual content in each film was timed in seconds. Sexual content was defined as romantic kissing, petting, and any form of intercourse (actual or implied). Implied sex could include such things as cars rocking and sexual noises as long as the sexual nature was clear. It did not include situations where there was no clear verbal or visual reference to sex (for instance, a couple leaving a bedroom without cues to imply sexual interaction). Each film was also rated in four ways: (1) salience of sexual behaviour (how “salient or noticeable was the sex?”), coded on a 5 point scale where 0 = no sex, 1 = not at all salient, 2 = minimally salient, 3 = moderately salient, 4 = extremely salient; (2) female nudity, coded on a 4-point scale where 0 = no female nudity, 1 = bare buttocks, 2 = bare breasts, 3 = full frontal female nudity; (3) male nudity, coded on a 3-point scale where 0 = no male nudity, 1 = bare buttocks, 2 = full frontal nudity; and (4) romance (“how romantic was the theme/content of the film (boy meets girl story, falling in love, building a relationship, “chic flick”, etc)?”), coded on a 4-point scale where 0 = no romantic content, 1 = minimally romantic content, 2 = moderate romantic content, 3 = extreme romantic content. Exploration of this additional information on salience, nudity and romance both independently and in combination showed that it did not add to the predictive power of timing of sexual content. Each film was rated by one of two trained coders, and a random sub-sample of 10% of films was double-coded (inter-rater agreement: *r* = 0.92). Out of any randomly selected 50 film titles, on average 36 (SD 3) had some sexual content although this varied according to the rating, with U and PG films averaging 8.6 (SD 12.6) s, 12 and 12A films 29.3 (SD 31.6) s, and 15 and 18 films 39.5 (SD 58.8) s each.

Pupils indicated the number of times they had ever seen each film on their unique list of 50 films (coded on a 5-point scale: 0, 1, 2, 3–5, 6+) to capture lifetime repeat viewing. We calculated **a duration measure of sexual content**: the total number of seconds of sexual content in all films watched allowing for the number of repeat viewings. This measure was divided into quartiles.

Three measures of **TV/DVD co-viewing frequency (with parents, same-sex peers or mixed-sex peers)** were each derived from single items asking teenagers how often they watched TV or DVDs with “your Mum or Dad (people that look after you at home)”, with “same sex friends”, and with “mixed sex friends”. Responses used five-point scales: every day, most days, weekly, less often and never/not applicable. In each case, three-point measures grouped the first two categories and the last two categories and were scored to run from low to high frequency of co-viewing. A measure of **parental share of TV/DVD co-viewing** was calculated from the difference between the parental co-viewing score and peer co-viewing score, where the latter was based on both same-sex and mixed-sex friends (using the more frequent response). A binary measure contrasted teenagers who watched TV/DVDs more often with friends and teenagers who were just as likely, or more likely, to watch with their parents than with friends.

**Parental media restrictions** were measured using questions about restrictions on watching TV/DVDs or playing on games consoles. A **restriction on sexual content** was based on agreement with either or both of two items: “I am not allowed to watch TV/DVDs or play games that contain a lot of nudity”; and “I am not allowed to watch TV/DVDs or play games with a lot of sexual activity in them”. **Other restrictions on media content** were measured using either or both of 2 items: no agreement with “I can watch anything I like”; and agreement with “I am not allowed to watch TV/DVDs or play games with a lot of violence”. **Restrictions on the duration or timing of media use in relation to other tasks** were measured using agreement with either or both of “I'm only allowed to watch a certain amount of TV/DVDs, or play games for a certain amount of time”; and “I have to finish my homework or special jobs first”.

### Covariates

Measures of overall exposure to films, television and cinema included: weekly hours watching TV or DVDs; frequency of cinema trips; and exposure to box office hits with a low sexual content (viewing of films rated U or PG). Further details of these measures are provided in Table 1.

Additional covariates included socio-demographic information, general parenting processes, school leaving plans, religiosity, sensation-seeking, self-esteem, malaise, friends' risk behaviours, the teenager's other risk behaviour (truancy and substance use), and whether the teenager had a boy/girlfriend. Details of measures used and sample information are provided in Table 1.

### Analysis sample

Film viewing information was only supplied by 2335 (59%) of pupils who filled in questionnaires, due to placement of the film viewing questions at the end of the questionnaire and the limited time available to answer questions (typically, one school lesson period). Completion of film data varied by year (47% in 2008 vs. 70% in 2009), school (ranging from 35% to 79%), gender (64% girls vs. 54% boys), and intention to stay on at school past the minimum school leaving age (64% amongst those intending to stay on vs. 40% of those planning to leave). Completion was also more likely for teenagers reporting lower involvement in risk behaviours (truancy, substance use and sexual behaviour). Of particular relevance here is the difference in completion according to sexual experience: 54% of those reporting sexual debut completed film data, compared to 67% without sexual experience. The film questions came immediately after questions on other important measures, including parental restrictions on media use and TV/DVD viewing hours, and missing information on these followed a similar pattern.

The eligible sample for analysis was restricted to cases with complete film viewing information. Within this sample, missing information levels were generally low, ranging between zero and 3% for individual measures; with the exceptions of teenager's school leaving plans (11%), mother's age (15%) and father's education (31%). The percentage of cases with missing information on any variable was 38%. To reduce bias and increase statistical power, multiple imputation using chained equations was used to impute 40 sets of data, up to the eligible sample (*n* = 2335) (White, Royston, & Wood, 2011). In principle it would have been possible to impute missing sexual film content in addition to other missing information, but imputation of such a large fraction of missing data would have been more sensitive to departures from the missing at random assumption (White et al., 2011). Analysis was restricted to cases with complete information for the outcome variable, sexual intercourse (*n* = 2251) (White et al., 2011).

### Analysis

The analysis first explored bivariate associations between media exposure measures, covariates, and sexual intercourse. Only covariates with a statistically significant (*p* < 0.05) association with sexual intercourse were included in multivariable models. Initial analyses were based on cases with complete information, but since these were similar to analysis using the imputed data set only the latter is shown here. All analyses were conducted using STATA 12.1 SE (Stata Corporation, Texas) and clustered responses by school.

## Results

The mean age of teenagers in the analysis sample was 15 years 6 months (range 14 years 8 months–16 years 11 months). Almost a third of teenagers (*n* = 733) reported intercourse (Table 2), with 58% of these experiencing intercourse before age 15. Out of the random selection of 50 blockbuster films, the mean number of films viewed was 20.0 (SD 7.5), including on average 5.5 (SD 3.5) films rated 15 and 18. The mean number of viewings per film was 2.1 (SD 0.7). Teenagers' mean total sexual content exposure for the random selection of 50 films was 18 min (1089, SD 836 s), allowing for repeat viewings. Four in ten teenagers watched TV/DVDs with their parents “every day” or “most days”, and around a quarter watched “every” or “most days” with same or mixed-sex friends. Around three in ten teenagers reported some restriction on the total amount or timing of media use in relation to other tasks such as homework. Only 13% reported a restriction on sexual content, although 21% reported other restrictions on media content including restrictions on violence. Table 2 presents further information on media exposure measures in relation to intercourse experience.

There were associations between many variables, as indicated by the correlation matrix in Table 3. Due to the large number of possible associations, only coefficients attaining statistical significance with *p* < 0.001 have been shown here. Intercourse was associated with exposure to sexual film content (coefficient 0.17), co-viewing TV/DVDs with same-sex friends (0.08) and with mixed-sex friends (0.29). Intercourse had negative associations with parental co-viewing and media restrictions (coefficients −0.10 to −0.14). Intercourse was associated with many other socio-demographic, family and teenage characteristics, in particular the teenager's own other risk behaviour, friends' risk behaviours and boy/girlfriend status (coefficients 0.45–0.49). Turning to associations among the main media exposure measures, teenagers who watched TV/DVDs with friends had higher exposure to sexual film content (coefficients 0.16 and 0.20 for same-sex and mixed-sex friends respectively); but there was no association between parental co-viewing and sexual film content. Parental restrictions on sexual media content were associated with lower sexual film exposure and mixed-sex peer TV/DVD co-viewing (both coefficients −0.09); and with more parental co-viewing (0.12).

**Fig. 1:**
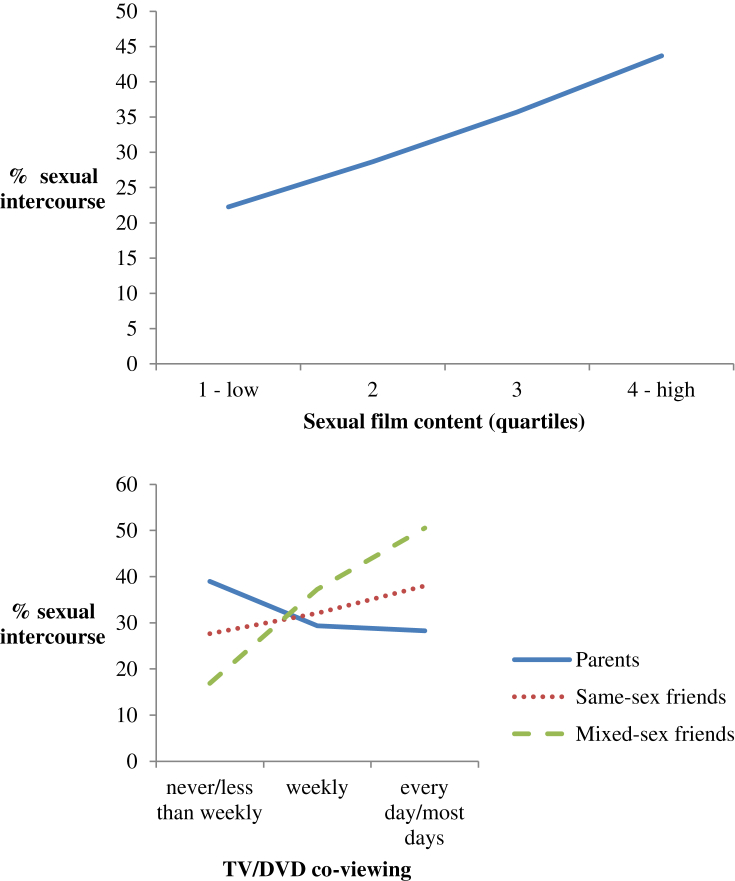
Univariate associations between media exposure measures and rates of sexual intercourse.

Fig. 1 shows univariate associations between the media exposure measures and sexual intercourse. There was a linear increase in the percentage reporting sexual intercourse with increasing sexual film content exposure. Parental co-viewing at least weekly was associated with a lower rate of sexual intercourse compared to viewing less often; while more frequent co-viewing with friends, especially mixed-sex friends, was associated with higher rates of sexual intercourse. Parental restrictions on media use, especially restrictions on sexual content, were associated with lower rates of intercourse. Unadjusted odds of sexual intercourse for these media exposure measures are given in the first column of figures in Table 4. This table also shows the reduced odds of intercourse associated with greater parental share of TV/DVD co-viewing.

Two adjusted models both controlled for socio-demographic, family and teenager characteristics (see Table 4 footnote for full list of covariates), as well as mutually adjusting for media exposures. The first adjusted model in Table 4 examined associations between sexual intercourse and sexual film content, parental media restrictions, frequency of parental co-viewing and parental share of co-viewing. In this model, the highest quartile of sexual film content exposure was associated with a 35% increase in the likelihood of intercourse compared to the lowest quartile, while a restriction on sexual media content and a greater parental share of TV/DVD co-viewing were associated with a reduced likelihood of intercourse (odds 0.68 and 0.74 respectively). The absolute frequency of parental co-viewing and other parental media restrictions had no significant associations with sexual intercourse. The second adjusted model replaced parental share of TV/DVD co-viewing with the two measures of peer co-viewing frequency (with same- and with mixed-sex friends). In this model, TV/DVD co-viewing with mixed-sex friends weekly or more often was associated with increased risk of intercourse (odds 1.85 and 2.61 respectively), while viewing daily with same-sex friends had the reverse association (odds 0.66). Sexual film content duration no longer had a significant association with intercourse, but the effect of parental restrictions on sexual content remained similar to the first model (odds 0.70). In this third model, other covariates that had independent statistically significant associations (*p* < 0.05) with intercourse were having a previous or current boy/girlfriend, the teenager's own other risk behaviour, friends' risk behaviour, a lack of religious beliefs and having more spending money (not shown).

Gender x media exposure interaction terms were added to test for gender differences in the effects of sexual film content, sexual media restriction, parental co-viewing share, and co-viewing with same- or mixed-sex friends. None was statistically significant. Pair-wise interactions amongst these media exposure measures were also explored, but again none was statistically significant. This suggested that the effect of sexual film content did not vary according to the context of co-viewing, and did not depend on parental media restrictions. Likewise, the effect of co-viewing did not seem to vary according to parental media restrictions.

## Discussion

This study found that teenagers who said that they watched TV/DVDs with friends more often than with parents were more likely to report sexual intercourse. Whereas co-viewing of TV/DVDs with same-sex friends was associated with lower risk for sexual intercourse, frequent co-viewing with mixed-sex friends was a particularly strong risk factor. We also found that the minority of teenagers who reported parental restrictions on sexual content were less likely to report intercourse than their peers. In contrast, frequency of parental co-viewing TV/DVDs and sexual film content were not associated with sexual intercourse after controlling for how television was watched.

The finding for parental restriction echoes other research suggesting parental restrictions may have protective effects for sexual behaviour (Ashby et al., 2006; Bersamin et al., 2008; Fisher et al., 2009) and other risk behaviours such as smoking and alcohol consumption (Dalton et al., 2006; Sargent et al., 2004; Tanski, Dal Cin, Stoolmiller, & Sargent, 2010). It adds to existing research by suggesting that specific restrictions on sexual content were more important for sexual behaviour than other restrictions on media content or use, although the cross-sectional nature of this study means that the direction of causation from parental restriction of sexual content to the young person's own sexual behaviour is uncertain: it is known that teenagers engaging in sensation-seeking and problem behaviours may elicit less restrictive parenting (de Leeuw et al., 2011; Racz & McMahon, 2011). The media variables included in the study could involve complex mediational pathways. Thus, parental restrictions on media content may have affected sexual behaviour indirectly by reducing exposure to sexual content, by reducing attention and importance attached to sexual content (Nathanson, 1999) and by lowering sensation-seeking (de Leeuw et al., 2011), even though the association for parental restrictions on sexual content found in our cross-sectional study was net of film sexual content exposure, perceived parental values and sensation-seeking. Parental restrictions on sexual content of TV, DVDs and electronic games may have also affected a broader range of media sexual content than captured in our measure of sexual film content exposure (see below); although the extent to which parents attempted or were able to enforce restrictions, particularly with high rates of bedroom media access (Bovill & Livingstone, 2001; Rideout et al., 2010), is unknown.

The absence of an association between more frequent parental co-viewing of TV/DVDs and sexual intercourse adds to limited existing research reporting mixed findings for the effects of parental co-viewing (Bersamin et al., 2008; Fisher et al., 2009). Effects of parental co-viewing may be subsumed in the effects of other family processes such as family time and connection (Padilla-Walker, Coyne, & Fraser, 2012). It is also possible that parental co-viewing conceals two opposing processes: increased “active” parental mediation through discussion of TV/DVD content for some teenagers, and greater perceived parental endorsement of sexual content for other teenagers whose parents do not express disapproval. Unfortunately our study did not measure active mediation so we are unable to disentangle these two processes, although existing research has not found associations between active mediation and sexual behaviour in teenagers (Bersamin et al., 2008; Fisher et al., 2009). It may be that the role of active parental mediation for teenagers is weaker than for younger children, reflecting decreased conformity with parents and increased conformity with peer attitudes during the mid teens (Berndt, 1979).

The findings for peer co-viewing extend limited previous evidence that peer co-viewing is associated with more favourable peer and individual orientation towards violent and sexual media content (Nathanson, 2001c). We lack detailed information on peer group composition; and it is as well to remember that with today's technology, the physical presence of peers is not always required as teenagers “multi-task” while viewing TV and contact friends via the Internet and mobile phones (Adriaens et al., 2011). However, watching (and possibly discussing) sexual content of TV programmes and DVDs with peers may increase their impact on teenagers (Rubin, 1993); and the presence of opposite-sex friends might strengthen any media effects on perceived peer norms, positive sexual outcomes and safe-sex self-efficacy (Bleakley, Hennessy, & Fishbein, 2011; Bleakley, Hennessy, Fishbein, et al., 2011; Martino, Collins, Kanouse, Elliott, & Berry, 2005). Effects on peer norms may arise from presumed greater influence of the media on others, rather than self (Gunther & Storey, 2003; Milkie, 1999), with the presence of opposite-sex co-viewers perhaps encouraging a teenager to perceive the opposite sex as more receptive to heterosexual advances. Although it is normal for the number of friendships with the opposite sex to grow during the teenage years, a high proportion of opposite-sex friendships may reflect more risky friendships, especially for girls (Mrug, Borch, & Cillessen, 2011; Poulin & Pedersen, 2007). Co-viewing in mixed-sex groups may therefore encourage risk behaviours more generally, by strengthening relationships with high-risk friends and a sense of identification with their activities (Suess et al., 1998). This is suggested by a study of smoking initiation, which found effects of film depictions of smoking appeared to be partly mediated by greater affiliation with peers who smoked (Wills et al., 2007). Alternatively, associations between mixed-sex peer co-viewing and sexual intercourse might reflect reverse causation, with mixed-sex co-viewing being simply a marker for groups of teens also comfortable with discussing and engaging in sexual behaviour. This interpretation is bolstered by the finding that teens that tend to watch television in same-sex groups have *lower* adjusted risk of engaging in sexual intercourse.

After allowing for frequency of peer TV/DVD co-viewing, this study did not find an independent association between duration of sexual content in films and increased risk of sexual behaviour in fifteen-year olds. This contrasts with longitudinal studies of teenagers' sexual media diet (Bleakley et al., 2008; Brown & L'Engle, 2009; Brown et al., 2006; Collins et al., 2004; Martino et al., 2006). The difference might reflect our study's focus on a narrower component of sexual media diet than other research looking at TV programmes, magazines, music and video games as well as films (Bleakley et al., 2008; Brown & L'Engle, 2009; Brown et al., 2006). In failing to capture these other venues, our study may have captured a relatively small proportion of total sexual media exposure. Some studies finding an effect of sexual media content also used broader approaches to sexual content analysis, including sex talk as well as behaviour (Bleakley et al., 2008; Brown & L'Engle, 2009; Brown et al., 2006; Collins et al., 2004). Another longitudinal study using similar methodology to our own in focusing on sexual behaviour in film content did find an effect of sexual content of films on reduced age of sexual initiation (O'Hara et al., 2012). The latter study did not, however, take account of peer risk behaviour, peer co-viewing, a teenager's other risk behaviour and boy/girlfriend status, all of which were associated with both sexual film content exposure and sexual behaviour in our data set. That study also modelled somewhat different outcomes (risky sex) and included mediational pathways from media sex exposure to risky sex through earlier onset sex and growth in sensation seeking, something we did not attempt in this cross-sectional study. As a result, causation in the present study is more uncertain: teenagers with risky friendships or a romantic partner may be more likely to seek out sexual media content (Bleakley, Hennessy, & Fishbein, 2011; Bleakley et al., 2008). Alternatively, formation of risky friendships, finding a boyfriend/girlfriend and co-viewing with mixed-sex friends might mediate associations between exposure to sexual film content and sexual intercourse, since exposure to such content may increase sensation-seeking (O'Hara et al., 2012). In allowing for these influences, we may have obscured indirect effects of sexual media exposure. These pathways need to be explored in future longitudinal studies.

Other limitations of this study include its reliance on self-reported data for sensitive information, although one study indicated a high validity of child-reported parental monitoring of media use (Gentile, Nathanson, Rasmussen, Reimer, & Walsh, 2012). We had no data on sexual media exposure from sources other than films, and the pattern of the high proportion of missing data on film exposure within our sample may affect the generalisability of the results to other populations. In contrast to studies directed at analysing differences in sexual content of TV and films according to genre (Fisher et al., 2009) or trends over time (Kunkel et al., 2007), we did not grade content according to the level of sexual behaviour (for example, by according sexual intercourse a higher score than kissing), although we found that our additional information on salience, romantic theme and nudity was redundant. However, our approach in assigning different sexual behaviours equal weight is similar to other studies exploring associations between sexual content and sexual behaviour (Bleakley et al., 2008; Brown & L'Engle, 2009; Brown et al., 2006; Collins et al., 2004; Martino et al., 2006; O'Hara et al., 2012). Before attempting to assess differential effects of various forms of sexual media content on adolescent behaviour, further research is needed to reconcile adolescent and researcher perceptions of sexual information. These show the strongest agreement for manifest content (Manganello et al., 2010).

We did not find gender differences in the effects of media exposure measures, unlike some other studies (Collins et al., 2004; Martino et al., 2009), and our predominantly white sample precluded investigation of ethnic differences found elsewhere (Brown et al., 2006). Differences in coverage between our sexual media exposure and contextual measures might explain why we did not find differential effects of sexual film content according to co-viewing or parental restrictions on media use, although other research also failed to find interactions between TV sexual content and various forms of parental mediation of TV (Fisher et al., 2009). Strengths include the ability to investigate under-explored aspects of media co-viewing with peers and specific parental rules for media use, while adjusting for more general parenting processes and aspects of teenage risk involvement.

While more research using longitudinal data is required to unravel causal processes, our findings suggest that future studies of the influence of sexual media content should take greater account of the context as well as the extent of sexual media exposure. For media consumption in the family environment, specific parental restrictions of sexual media content may be a more effective strategy to protect teenagers from early sex than parental co-viewing. Caution is required, since restrictions in the absence of discussion and trust may not be effective (Nathanson, 2002). Further work is needed to examine possible differences in the effectiveness of parental mediation strategies according to the child's age (Nathanson, 2001c), especially since exposure to sexual content may begin well before the teenage years and then be actively sought out with the onset of puberty (Brown, Halpern, & L'Engle, 2005). Some research has found promising effects of media literacy programmes (Pinkleton, Austin, Chen, & Cohen, 2012; Pinkleton, Austin, Cohen, Chen, & Fitzgerald, 2008), and this study suggests that the success of these may be improved with a greater understanding of how social interaction with peers may both shape exposure to sexual material, and influence how such material is experienced.

## Figures and Tables

**Table 1:** Socio-demographic information, general media exposure, parenting and teenager characteristics.

Variable	Description	Range	% or Mean (SD)
Gender	Gender	Male = 0, Female = 1	52% female
Age	Age in years	14.7–16.9 years	15.5 (0.3)
Father's education	Father continued with education past 16 years	0 = stayed on post-16 years, 1 = left school at or before 16 yrs	51% left school by 16 yrs
Mother's age	Mother's age at time of survey	0 = 40 years or older, 1 = under 40 years	21% under 40 years
Family type	Number of biological parents in household	0 = lives with both parents, 1 = lives with lone parent or in a reconstituted family	32% live with lone parent or in reconstituted family
School leaving plans	Number of years of secondary education intends to complete	0 = plans to complete less than 5 years, 1 = plans to complete 5 years, 2 = plans to complete 6 years	16% plan to complete 5 years, 68% plan to complete 6 years
Religiosity	Extent of religious belief	1 “not at all religious” to 5 “very religious”	46% some religious belief (score>1)
Year	Survey conducted in 2008 or 2009	2008 = 1, 2009 = 2	59% in 2009
Area	Area of Scotland	East of Scotland = 1, West of Scotland = 2	60% West of Scotland
Exposure to U and PG blockbuster films	Proportion of U and PG films asked about that teenager has viewed	0 to 1	0.44 (0.19)
TV/DVD viewing hours	Weekly hours estimated using two items about time spent watching TV/DVDs on an average school day and on an average weekend day	3.5 to 67	18.35 (10.94)
Cinema visits	How often goes to cinema	0 “less than once a week”, 1 “weekly”, 2 “more than once a week”	40% weekly, 6% more than once a week
Parental monitoring.	Four items (Cronbach alpha = 0.62) about rules for going out in spare time (being back by a certain time, someone stays up until teenager gets home, having to phone or text parents if teenager changes plans, and whether parents really know where teenager is)	1 “always” to 4 “never”. Reverse coded for analysis so high score = greater monitoring	1.98 (0.61)
Pocket money	Single item asking how much money teenager has each week to spend as they like	1 “nothing” to 7 “£30 or more”	4.11 (1.65)
Parental supportiveness	Two items (Pearson *r* = 0.51) asking for agreement with "My parents/guardians…sense when I'm upset about something", "…encourage me to talk about my difficulties"	1 “strongly agree” to 5 “strongly disagree”	2.27 (0.87)
Parental psychological control	Two items (Pearson *r* = 0.59) asking for agreement with "My parents/guardians try to control everything I do", "…treat me like a baby"	1 “strongly agree” to 5 “strongly disagree”	3.54 (1.04)
Disagreements with parents	Single item asking about the frequency of serious disagreements or arguments about things (for instance drinking, your friends, homework, tidiness or what you wear)	1 “every day” to 5 “never”	3.23 (1.04)
Parental attitudes to teen sex	Four items (Cronbach alpha = 0.72) on whether each parent/guardian "thinks people should be in a loving relationship before having penetrative sex", "would disapprove of me having penetrative sex". For 2 parents, mean scores were used	1 “strongly agree” to 5 “strongly disagree”	2.55 (0.72)
Family time	Four items (Cronbach alpha = 0.72) about frequency of eating together, going for a walk or playing sport together, going places together or doing other things as a family group	1 “every day” to 5 “never”	3.24 (0.68)
Sensation-seeking	Four items, from the Brief Sensation Seeking Scale, BSSS-4 (Stephenson, Hoyle, Palmgreen, & Slater, 2003) (Cronbach alpha = 0.71) (“I would like to explore strange places”, “I like to do frightening things”, “I like new and exciting experiences, even if I have to break the rules”, “I prefer friends who are exciting and unpredictable”)	1 “strongly agree” to 5 “strongly disagree”	2.43 (0.71)
Self-esteem	Three items (Cronbach alpha = 0.72) "I like myself", "I am a failure" (reversed), "Most of the time I am satisfied with the way I look"	1 “strongly agree” to 5 “strongly disagree”	1.93 (0.51)
Malaise	Six items (Cronbach alpha = 0.74) taken from the 12-item General Health Questionnaire (Tait, French, & Hulse, 2003) about whether teenager has in the past 2 months 1) been able to concentrate, 2) felt capable about making decisions, 3) been able to enjoy normal activities, 4) been losing confidence, 5) thinking of self as a worthless person, 6) been feeling reasonably happy	Items 1–3 and 6: 1 “better than usual” to 4 “much less than usual”. Items 4 and 5: 1 “not at all” to 4 “much more than usual”	1.92 (0.50)
Friends' risk behaviour	Four items (Cronbach alpha = 0.80) about the proportion of the teenager's friends who had left school, who smoked most days, who got drunk most weeks, or had experienced sex	1 “none” to 5 “all”	2.11 (0.81)
Own other risk behaviour	Four items (Cronbach alpha = 0.74) about frequency of skipping school, getting drunk, smoking, and using cannabis	Skipping school 1 “strongly agree" to 5 “strongly disagree” (reverse-coded). Substance use 1 “never” to 6 “more than once a week”	2.00 (1.03)
Partner status	Teenager has a boyfriend or girlfriend	0 = never, 1 = used to have one, but not now, 2 = have one now	50% used to have a boyfriend/girlfriend, 31% have a current boyfriend/girlfriend

**Table 2:** Main media exposures for whole sample and according to main sexual outcome measures.

		All	Intercourse	
			No	Yes
*N* (row %)		2335 (100)	1518 (67.4)	733 (32.6)
**Sexual film content exposure**				
Seconds	Mean (SD)	1089.5 (835.8)	991.6 (763.5)	1296.4 (951.6)
	Median (interquartile range)	905 (530–1430)	818 (474, 1321)	1076 (662, 1701)
**Co-viewing TV/DVDs**				
Parents	Every day/most days	39.2	41.8	34.3
	Weekly	24.1	24.9	21.5
	Less often/never	36.7	33.4	44.2
Same-sex friends	Every day/most days	28.7	26.4	33.6
	Weekly	42.2	42.4	41.6
	Less often/never	29.2	31.2	24.8
Mixed-sex friends	Every day/most days	24.9	18.3	39.1
	Weekly	35.0	32.3	40.0
	Less often/never	40.1	49.3	20.9
Parental share of co-viewing	Friends more than parents	37.5	31.8	49.9
	Parents same as/more than friends	62.5	68.3	50.1
**Parental restrictions on TV/DVD/electronic game use**				
Restriction on duration/timing in relation to tasks	No (%)	68.7	64.8	75.9
	Yes (%)	31.3	35.2	24.2
Restriction on sexual content	No (%)	86.9	83.6	93.6
	Yes (%)	13.1	16.4	6.4
Other content restriction	No (%)	78.7	75.9	84.6
	Yes (%)	21.3	24.1	15.4

**Table 3:**
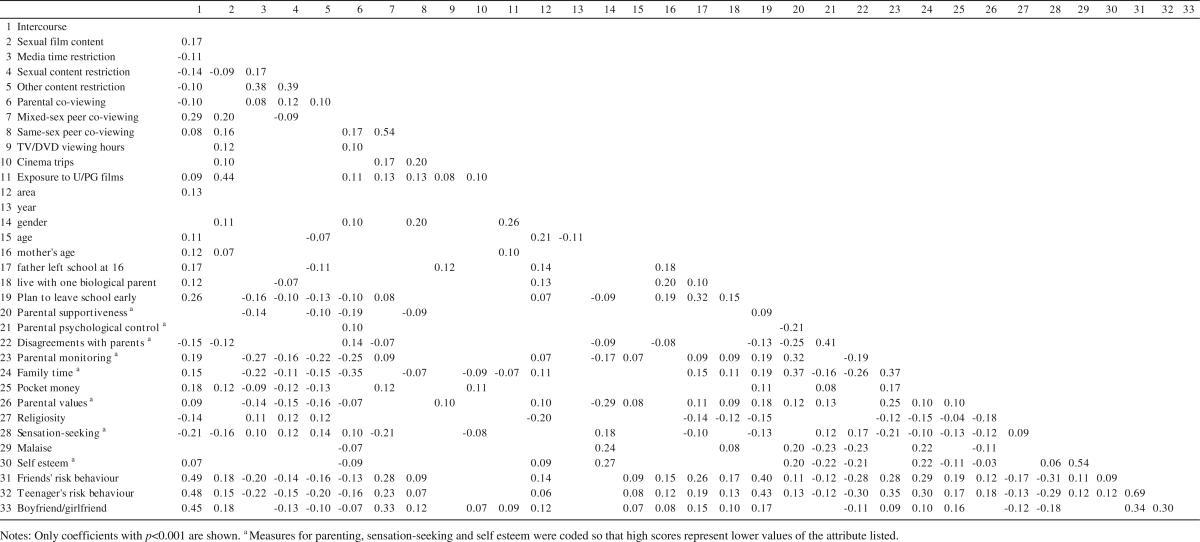
Zero-order correlations between study variables.

**Table 4:** Associations between media exposure measures and sexual intercourse: results of logistic regression analyses.

Imputed data set *N* = 2335		Unadjusted	*p*	Adjusted model (1)	*p*	Adjusted model (2)	*p*
		OR (95% CI)		OR (95% CI)		OR (95% CI)	
Sexual film content (lowest quartile)	q2	1.40 (1.06, 1.87)	0.020	0.98 (0.73, 1.30)	0.866	0.95 (0.71, 1.28)	0.755
	q3	1.94 (1.51, 2.50)	<0.001	1.14 (0.90, 1.44)	0.268	1.10 (0.87, 1.38)	0.430
	q4 – Highest quartile	2.71 (2.07, 3.56)	<0.001	1.35 (1.00, 1.82)	0.046	1.30 (0.93, 1.80)	0.120
*Parental media restrictions*							
Restriction on duration/timing in relation to homework or household tasks (none)	Yes	0.59 (0.47, 0.73)	<0.001	1.07 (0.78, 1.46)	0.691	1.06 (0.77, 1.47)	0.720
Restriction on content (none)	Sexual content restriction	0.33 (0.28, 0.39)	<0.001	0.68 (0.48, 0.96)	0.029	0.70 (0.50, 0.98)	0.039
	Other content restriction	0.68 (0.50, 0.94)	0.018	1.10 (0.76, 1.60)	0.611	1.09 (0.76, 1.56)	0.636
Parental share of co-viewing (friends more than parents)	Parents same as/more than friends	0.47 (0.38, 0.58)	<0.001	0.74 (0.58, 0.93)	0.011	_	
TV/DVD co-viewing with parents (never/less than weekly)	Weekly	0.65 (0.49, 0.87)	0.004	1.16 (0.79, 1.70)	0.455	1.02 (0.74, 1.42)	0.884
	Every day/most days	0.62 (0.49, 0.77)	<0.001	1.14 (0.76, 1.72)	0.537	0.90 (0.65, 1.24)	0.523
TV/DVD co-viewing with same-sex friends (never/less than weekly)	Weekly	1.24 (1.04, 1.49)	0.019	_		0.90 (0.62, 1.29)	0.560
	Every day/most days	1.60 (1.33, 1.93)	<0.001	_		0.66 (0.45, 0.96)	0.032
TV/DVD co-viewing with mixed-sex friends (never/less than weekly)	Weekly	2.91 (2.37, 3.58)	<0.001	_		1.85 (1.18, 2.92)	0.008
	Every day/most days	5.06 (4.02, 6.37)	<0.001	_		2.61 (1.74, 3.92)	<0.001

Notes: unadjusted models show individual associations between each media exposure measure and sexual intercourse (measures are not mutually adjusted). Adjusted models controlled for share of U/PG films viewed, area, gender, age, mother's age, father left school at 16, live in one-parent or reconstituted family, school leaving plans, parental psychological control, disagreements with parents, parental monitoring, family time, pocket money, parental values, religiosity, malaise, self-esteem, sensation-seeking, friends' risk behaviours, the teenager's other risk behaviour and boy/girlfriend status. Media exposure measures are all mutually adjusted.
